# Transforming activity of an oncoprotein-encoding circular RNA from human papillomavirus

**DOI:** 10.1038/s41467-019-10246-5

**Published:** 2019-05-24

**Authors:** Jiawei Zhao, Eunice E. Lee, Jiwoong Kim, Rong Yang, Bahir Chamseddin, Chunyang Ni, Elona Gusho, Yang Xie, Cheng-Ming Chiang, Michael Buszczak, Xiaowei Zhan, Laimonis Laimins, Richard C. Wang

**Affiliations:** 1 Department of Dermatology, UT Southwestern Medical Center, Dallas, TX 75390 USA; 2 Quantitative Biomedical Research Center, UT Southwestern Medical Center, Dallas, TX 75390 USA; 3 Department of Molecular Biology, UT Southwestern Medical Center, Dallas, TX 75390 USA; 4 Department of Microbiology-Immunology, Feinberg School of Medicine, Northwestern University, Chicago, IL 60611 USA; 5 Simmons Comprehensive Cancer Center, UT Southwestern Medical Center, Dallas, TX 75390 USA; 6 Department of Biochemistry, UT Southwestern Medical Center, Dallas, TX 75390 USA; 7 Department of Pharmacology, UT Southwestern Medical Center, Dallas, TX 75390 USA

**Keywords:** Tumour virus infections, Human papilloma virus, RNA metabolism

## Abstract

Single-stranded circular RNAs (circRNAs), generated through ‘backsplicing’, occur more extensively than initially anticipated. The possible functions of the vast majority of circRNAs remain unknown. Virus-derived circRNAs have recently been described in gamma-herpesviruses. We report that oncogenic human papillomaviruses (HPVs) generate circRNAs, some of which encompass the E7 oncogene (circE7). HPV16 circE7 is detectable by both inverse RT-PCR and northern blotting of HPV16-transformed cells. CircE7 is N^6^-methyladenosine (m^6^A) modified, preferentially localized to the cytoplasm, associated with polysomes, and translated to produce E7 oncoprotein. Specific disruption of circE7 in CaSki cervical carcinoma cells reduces E7 protein levels and inhibits cancer cell growth both in vitro and in tumor xenografts. CircE7 is present in TCGA RNA-Seq data from HPV-positive cancers and in cell lines with only episomal HPVs. These results provide evidence that virus-derived, protein-encoding circular RNAs are biologically functional and linked to the transforming properties of some HPV.

## Abstract (web summary)

The authors identify circular RNAs (circRNA) from human papillomavirus and show that circRNA-encoded E7 contributes to cancer cell growth in vitro and in tumor xenografts. Furthermore, circE7 is present in TCGA RNA-Seq data from HPV-positive cancers.

## Introduction

Human papillomaviruses (HPVs) are small, double-stranded DNA viruses that infect stratified epithelia. While the majority of HPV infections are asymptomatic or cause benign warty growths, a subset of ‘high-risk’ HPVs can promote the development of cervical, oropharyngeal, anal, vulvovaginal, and penile cancers. The recognition of the critical role of HPV infection in the pathogenesis of ~5% of human cancers has spurred the development of vaccines that have the potential to decrease the burden HPV-driven malignancies^1^. Despite this progress, our understanding of how high-risk HPVs progress from latent infections to incurable cancers remains incomplete.

One strategy employed by HPV to regulate its life cycle is through alternative splicing of its relatively small genome^2^. In particular, extensive splicing of the early region of high-risk HPV appears to be critical for its tumorigenic properties. The E6 and E7 oncoproteins are transcribed on a bicistronic mRNA, and most E7 oncoprotein translation occurs from a truncated E6 transcript (E6*I) through a mechanism involving translation reinitiation^3,4^. However, mutations abolishing the 5′ splice donor in the E6 intron do not completely abolish E7 oncoprotein expression suggesting that alternative, uncharacterized transcripts might also contribute to E7 translation^4,5^. In addition to the alternative splicing of mRNAs, many viruses employ additional strategies, like the production of non-coding RNA to promote their life cycle. Non-coding RNAs, like adenovirus VA and Epstein-Barr Virus (EBV) EBER, prevent the activation of innate immune responses^6^. Similarly, miRNA, like those encoded by EBV and the SV40 polyomavirus, have also been shown to limit activation of the host immune response through host or viral targeting^7,8^.

The occurrence of covalently closed single-stranded RNAs (circRNAs) was initially thought to be limited to viroids and rare splicing events from uncommon loci^9^. However, with the recent realization that circRNAs are abundant, interest in this class of RNA molecules has increased. The best characterized circRNAs function as microRNA sponges. For example, circHIPK3, a circRNA derived from the second exon of *HIPK3*, binds multiple miRNAs, including miR-124, to promote cancer cell growth in vitro^10^. However, recent studies have suggested additional roles for circRNAs, including the ability of some circRNAs to code for proteins. Because the translation of circRNAs appears to be markedly lower than that of 5′, 7-methylguanosine capped and polyadenylated transcripts, the biological relevance of circRNA-driven protein production remains unclear. While recent studies have also revealed that EBV and Kaposi Sarcoma Virus (KSHV) generate a diverse menagerie of circRNA^11,12^, the functions of these viral circRNAs remain uncertain.

We report here the discovery of circRNAs from high-risk HPV. Characterization of the abundant HPV16 circE7 revealed that it can be translated through cap-independent mechanisms. HPV-derived circE7 is abundant in cervical and head and neck cancers in The Cancer Genome Atlas (TCGA), and HPV16 circE7 is essential for the transformed growth of CaSki cervical carcinoma cells.

## Results

### Identification and detection of HPV circRNAs

**Fig. 1:**
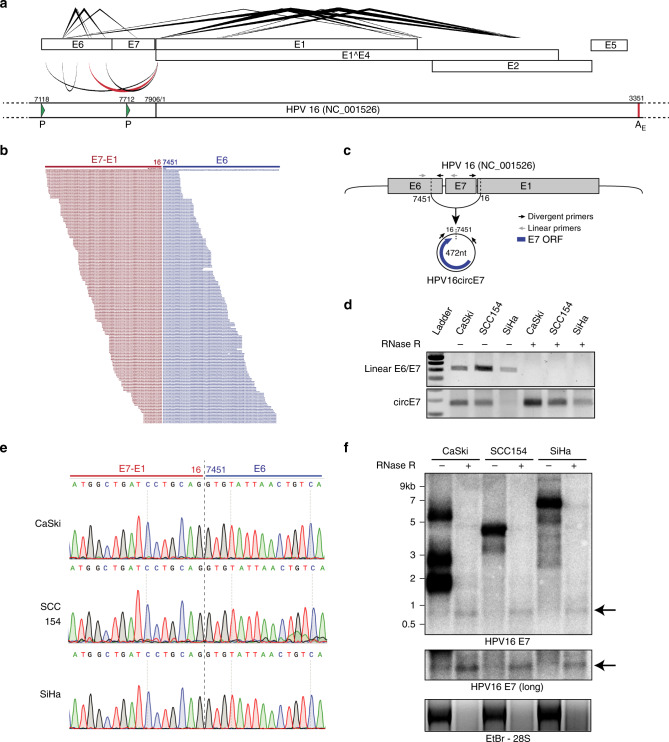
Identification of HPV circRNAs. **a** A transcript map generated by vircircRNA summarizing the splicing events identified for HPV16 from the combined SRA datasets (Supplementary Fig. 1e). Lines (top) indicate forward splicing events; arcs (bottom) indicate backsplicing; thickness = log_2_(read count); red arc highlights circE7. The lower panel represents a partial HPV16 genome with promoters (P, green arrowheads) and the early polyadenylation (A_E_, red line) indicated. Numbering from the NC_001526 reference sequence. **b** Alignment of sequencing reads spanning the circE7 backsplice junction from SRS2410540. Red indicates E7-E1 sequences, and blue indicates E6 sequence. **c** Predicted formation and size of HPV16 circE7. Arrows indicate primers used to detect linear E6/E7 and circE7. **d** RT-PCR of random hexamer primed total RNA from HPV16+ cancer cell lines. 2 μg of total RNA were treated with 5U of RNase R (or water for mock) in the presence of RNase inhibitor for 40 min prior to RT reaction. Results are representative of 4 independent experiments. **e** Sanger sequencing of PCR products from **d** confirmed the presence of the expected circE7 backsplice junction without the insertion of additional nucleotides. Sequencing traces were identical for 3 independent reactions from each cell line. **f** Northern blot of total RNA after mock (8 μg) or with RNase R treatment (20 μg) from the indicated HPV16+ cell line probed with HPV16 E7. Arrows indicates RNase resistant band with E7 sequence. Ethidium Bromide staining (bottom), RNase R treatment control. Results representative of 5 independent northerns

To screen for the presence of circRNA in HPV, we developed a pipeline to detect and visualize backsplice junctions from viral genomes (vircircRNA). To ensure accurate identification of all backsplices, circular viral genomes were concatenated and then utilized as the reference genome for the pipeline (Supplementary Fig. 1a–b). We selected ten HPV subtypes (Supplementary Fig. 1c) as reference genomes to screen against publicly deposited RNA-Seq datasets (Supplementary Fig. 1d). We identified 12 projects with RNA-Seq data from HPV-infected tissues. Despite the fact that most samples were not optimized for circular RNA sequencing through RNase R treatment or ribosomal RNA depletion, we identified 27 samples with multiple reads mapping to putative backsplice junctions (Supplementary Fig. 1e). In our initial screen, backsplice reads were identified from HPV16 and HPV35 (Fig. 1a, Supplementary Fig. 1e). Specific HPV16 circRNAs were notable because their abundance was comparable to spliced linear mRNAs (Fig. 1a, b). The vast majority (94%) of backsplice reads were generated from the head-to-tail joining of previously reported linear splice sites downstream in E1 (nt 16, E1^E4 splice donor) to one upstream in E6 (nt 7451, E6*I splice acceptor) (Fig. 1a–c, Supplementary Fig. 1e). This backsplicing is predicted to form a 472 nt circular RNA, which contains the entire open reading frame of E7 (Fig. 1c). Due to the established importance of the E7 oncogene and the abundance of HPV16 circE7, we focused on the characterization of this newly identified circRNA. We tested for the presence of backsplicing by inverse PCR using three cancer cell lines in which HPV16 has been shown to be stably integrated (CaSki and SiHa cervical cancer, UPCI-SCC154 tongue squamous cell cancer). RNase R is an exoribonuclease that specifically degrades linear, but not circular or lariat, RNAs. While a linear region of HPV16 E6/E7 was markedly decreased in abundance after RNase R treatment, the circE7 junction detected in all 3 cell lines before RNase R was enriched after treatment (Fig. 1d). Sanger sequencing of the amplified inverse PCR product from all three cell lines confirmed that the circE7 backsplice represented a true splice site rather than an intron lariat (Fig. 1e), which frequently contains untemplated nucleotides across the branch point^13^. HPV16 circE7 could be identified in the cancer cell lines by northern blotting as an RNase R-resistant band that migrated more slowly than its predicted size due to its circular structure^14^ (Fig. 1f). Quantitation of the northern blots revealed that circE7 represents ~1–3% of total E7 signals. Given the prevalence of HPV18 in HPV-induced cancers, we also tested 3 HPV18+ cell lines for the presence of a similar circular RNA (HPV18 circE7) (Supplementary Fig. 2a). While an analogous HPV18 circE7 could not be robustly detected by either RT-PCR or northern blotting (Supplementary Fig. 2b–c), RNA-Seq of SW-756 cervical carcinoma cells revealed several backsplice junction reads consistent with HPV18 circE7 as well as other potential circRNAs (Supplementary Fig. 2d).

### Formation and translation of circE7

**Fig. 2:**
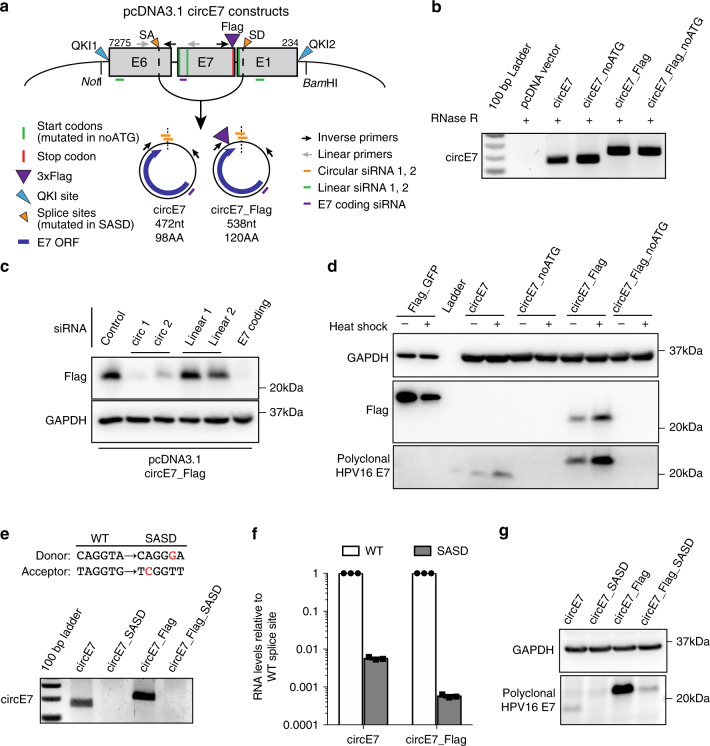
Generation and translation of circE7. **a** Diagram of constructs generated to express circE7 in vitro. Map indicates location of QKI sites, start codons (all mutated in ‘noATG’), 3xFLAG (present in ‘FLAG’), splice sites (mutated in ‘SASD’), and siRNA used in subsequent experiments. **b** RT-PCR confirms the formation of RNase R-resistant circE7 RNAs from transfected 293 T cells. Results representative of >6 independent experiments. **c** Western blot for FLAG from 293 T cells co-transfected with circE7_FLAG and the indicated siRNA. GAPDH, loading control. Results representative of 4 independent transfections. **d** Western blots for FLAG and HPV16 E7 from 293 T cells transfected with the indicated circE7_FLAG construct (4 μg) or a linear FLAG-GFP control vector (0.4 μg). Indicated transfections were subjected to a heat shock (2 h at 42 °C, 2 h recovery). Eight-fold less lysate was loaded for control FLAG-GFP transfected cells. GAPDH, loading control. Results representative of 5 independent experiments. **e** RT-PCR demonstrates that splice sites indicated in **a** are required for the formation of circE7 RNAs. **f** RT-qPCR from 293 T cells transfected with pcDNA3.1-circE7 constructs confirms significantly lower circRNA formation in the SASD mutants. Results representative of 3 independent experiments. **g** Western blots for HPV16 E7 from 293 T cells transfected with the indicated circE7 construct (4 μg). GAPDH, loading control. Source data for **b**, **d**, **e**, and **g** provided in Source Data file

CircRNAs have been reported to function by sponging miRNAs^15–17^. To determine whether circE7 might have a role as a miRNA sponge, we examined whether any miRNA-binding sites exist on the transcripts. While both HPV16 and HPV35 circE7s were predicted to have miRNA-binding sites (Supplementary Fig. 3a–b), none of the predicted miRNA-binding sites were conserved between the HPV16 and HPV35 circE7 species^18^. Since circRNAs have the potential to encode peptides, we next tested whether circE7 might be translated. To facilitate the detection of circE7 translation, we generated minigene expression vectors encompassing the entire ~1 kb backspliced region of the HPV16 genome (Fig. 2a). While the HPV sequence alone did promote circE7 generation, the addition of quaking (QKI) protein-binding sites, which have previously been reported to facilitate the circularization of RNAs^19^, enhanced E7 circularization by more than two-fold (Supplementary Fig. 3c). To determine whether circE7 had the potential to encode E7, constructs containing mutations of all three potential start codons (circE7_noATG) and E7 ORFs with a C-terminal 3xFLAG epitope tag were generated (Fig. 2a). Human embryonic kidney cells (HEK293T) were transfected with the minigenes and assayed for circRNA formation by RT-PCR using inverse primers. We detected RNase R-resistant circRNAs of the expected size from both WT and epitope-tagged circE7 (Fig. 2b). When HEK293T cells were transfected with the circE7 minigenes, we were able to detect E7 protein using both HPV16 E7-specific and FLAG antibodies, but not when the E7 start codons were mutated (Fig. 2c, d). The circE7 expression construct lacks the upstream E6 sequences required to generate any linear mRNAs previously reported to produce the E7 oncoprotein, including the E6*I isoform. To confirm that circular, rather than linear E7 RNAs, were responsible for E7 translation, we designed small interfering RNAs (siRNAs) to target sequences specific to the backsplice junction, the linear mRNA, or a region shared by both linear and circular species (Fig. 2a). RT-PCR confirmed that siRNAs against the circE7 backsplice preferentially knocked down the circular transcript; those against linear E6/E7 preferentially depleted the linear transcript; and those targeting E7 knocked down both RNAs (Supplementary Fig. 3e). Notably, knockdown of circE7 or shared regions of the E7 ORF inhibited the expression of E7 protein. In contrast, siRNAs targeting the linear RNA did not strongly decrease E7 expression by western blotting (Fig. 2c). CircRNAs are predicted to be translated through a cap-independent mechanism, which can be upregulated by cell stressors, including heat shock^20,21^. 293T cells transfected with wild-type or FLAG-tagged circE7 constructs increased E7 translation, greater than four-fold and two-fold, respectively, in response to a 42 °C heat shock (Fig. 2d, Supplementary Fig. 3f). In contrast, a linear control RNA (FLAG-GFP) showed a greater than two-fold decrease in expression after heat shock (Fig. 2d, Supplementary Fig. 3f). To determine whether conserved splice sites were necessary for circE7 formation and expression, a circE7 minigene with mutations in both the splice acceptor and splice donor sites was generated (Fig. 2a–e; Supplementary Data 2). As expected, the splice site mutant constructs (circE7_SASD, circE7_FLAG_SASD) showed significantly lower levels of circE7 by both end point and RT-qPCR (Fig. 2e, f). E7 expression was markedly reduced after splice site mutation providing further evidence that circE7, rather than linear mRNAs, provided the template for E7 oncoprotein translation (Fig. 2g).

### Molecular characterization of circE7

**Fig. 3:**
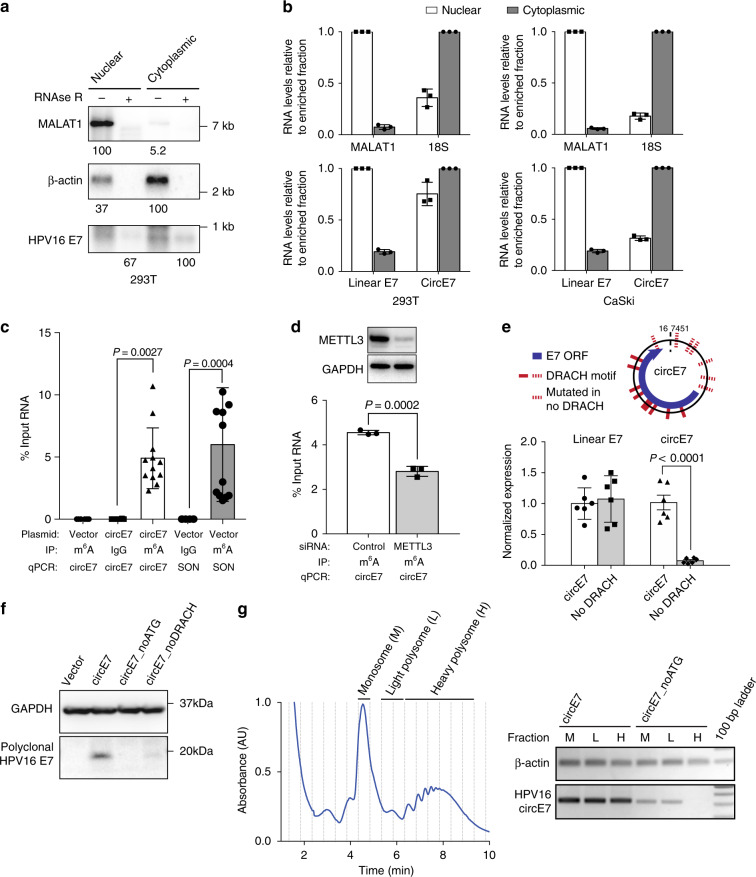
Characterization of circE7. **a** CircE7-transfected cells were fractionated and indicated fractions analyzed by northern blot. Total RNA (4 μg) with mock or RNase R treatment of fractions from 293 T cells confirms that circE7 is enriched in the cytoplasm and is RNase R-resistant. MALAT1 and β-actin, fractionation controls. Band density (bottom) quantitated after normalization to the enriched fraction. Results are representative of 3 independent blots. **b** CircE7-transfected 293T (left) or untransfected CaSki (right) were fractionated and analyzed by RT-qPCR. MALAT1 and 18 S (top), fractionation controls. Values normalized to the enriched fraction. Results are representative of 3 independent fractionation experiments. **c** RT-qPCR of RNA IP (m^6^A or IgG control) after transfection with the indicated plasmid (24 h) (*n* = 8 biological replicates from 4 transfections). SON, m^6^A RNA IP control. **d** Western blot for METTL3 from 293T co-transfected with control or METTL3 siRNA and circE7 construct (top). GAPDH, loading control. RT-qPCR of RNA IP (m^6^A or IgG control) from 293 T cells co-transfected with indicated siRNA and circE7 construct. RT-PCR is representative of 4 independent experiments. **e** Schematic of the DRACH consensus motifs for METTL3/14 and the sites mutated in the circE7_noDRACH construct (top). RT-qPCR for circE7 in cells transfected with the indicated construct. Loss of UTR DRACH motifs in circE7 results in a significant decrease in the abundance of circE7, but not linear E6/E7. (*n* = 4 independent experiments). **f** Western blot for E7 from 293 T transfected with indicated circE7 construct. Data are shown as mean ± s.d. *P* values (indicated above relevant comparisons) were calculated with one-way analysis of variance (ANOVA) with Holm–Sidak tests. **g** Representative tracing of circE7-transfected cells after polysome enrichment assay with the monosome (M), light polysome (L), and heavy polysome (H) fractions indicated (left). Dashed lines indicate collected fraction. Detection of circE7 in polysome fraction by RT-PCR after transfection with circE7 or circE7_noATG (right). β-actin, control. Source data for **a** provided in Source Data file

To determine the subcellular localization of circE7, cells were fractionated and nuclear and cytoplasmic fractions confirmed with MALAT1 and 18S or β-actin, respectively. Consistent with the behavior of other translated circRNAs^20,22–24^, northern blots revealed that the majority of circE7 localized to the cytoplasm (Fig. 3a), and RT-qPCR confirmed the cytoplasmic enrichment of circE7 in both transfected 293 T cells (~58%) and CaSki cells (~75%) (Fig. 3b). Cap-independent translation of circRNAs has been reported to require N^6^-methyladenosine (m^6^A) modifications in the UTR^20^. Because HPV16 circE7 possessed multiple potential m^6^A consensus sites (DRACH) (Fig. 3e), we performed m^6^A RNA immunoprecipitation (IP) experiments. Antibodies against m^6^A, but not an IgG control, pulled down circE7 with a comparable efficiency to SON, a control mRNA containing multiple m^6^A sites and previously confirmed to be methylated (Fig. 3c)^20^. Consistent with the previously described role for METTL3/14 in circRNA methylation, siRNA-mediated knockdown of METTL3 resulted in decreased recovery of circE7 after m^6^A IP (Fig. 3d). Next, we constructed a circE7 mutant in which potential m^6^A motifs in the UTR were mutated (circE7_noDRACH) (Fig. 3e). Unexpectedly, this construct dramatically decreased the abundance of circE7, but not its linear E6/E7 counterpart (Fig. 3e, Supplementary Fig. 3g). Mutation of the potential m^6^A motifs strongly inhibited E7 oncoprotein expression, once again confirming the critical role for the circular, rather than the linear, RNA in E7 translation (Fig. 3f). Finally, we performed polyribosome (polysome) fractionation to determine whether circE7 might be associated with polysomes as has been reported for other translated circRNAs^20,23^. Analysis of monosome (M), light polysome (L), and heavy polysome (H) fractions revealed circE7 to be associated with all fractions. Mutation of the start codons in circE7, circE7_noATG, abrogated the association of circE7 with heavy polysomes and attenuated its presence in the monosome and light polysome fractions. The association of a control mRNA (β-actin) was not affected (Fig. 3g). Thus, circE7 is m^6^A-modified, enriched in the cytoplasm, associated with polysomes, and capable of generating the E7 oncoprotein in a heat-shock regulated manner.

### Functional characterization of circE7 in cancer

**Fig. 4:**
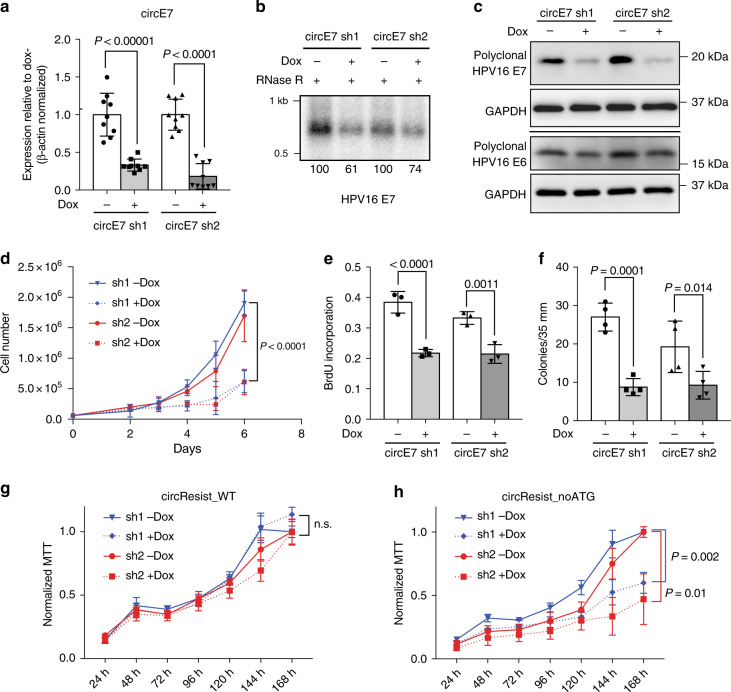
Protein encoding circE7 is essential for CaSki cell growth. **a** CaSki cells were lentivirally transduced with doxycycline (dox)-inducible hairpins specific for the circE7 backsplice junction (circE7 sh1/2). RT-qPCR for levels of circE7 revealed that circE7 sh1/2 resulted in significant decreases of circE7 levels. (*n* = 3 independent experiments, run in duplicate). **b** Northern blot of RNase R treated total RNA (30 μg) from CaSki cells with or without circE7 sh1/2 induction (2 days). Band density (bottom number) was quantitated and normalized to the uninduced control. **c** Western blots for E7 and E6 after circE7 sh1/2 induction (3 days). Western blots representative of 3 independent experiments. GAPDH, loading control. **d** A total of 6.0 × 10^4^ CaSki cells were seeded in triplicate in six-well plates at day 0 and absolute cell number quantitated daily after day 2. CircE7 sh1/2 induction resulted in significantly slower growth of CaSki cells after day 4. Similar results were obtained in 3 independent experiments. **e** CaSki cells with or without circE7 sh1/2 induction (1 day) were plated in chamber slides and labeled with BrdU (10 μM for 1.5 h). Cells were stained with αBrdU and DAPI and scored as % of DAPI + cells. **f** 1.0 × 10^4^ CaSki circE7 sh1/2 cells with or without induction (1 day) were seeded in triplicate in soft agar with or without dox (14 days). Average colonies per 35 mm. *n* = 4 independent transfections. **g** CaSki were doubly transduced with a shRNA resistant WT circE7 (circResist_WT) and circE7 sh1/2. MTT assay of circResist_WT cells with and without Dox induction. MTT values normalized to the uninduced (-Dox) condition. **h** CaSki were doubly transduced with a shRNA resistant circE7 with no start codons (circResist_noATG) and circE7 sh1/2. MTT assay of circResist_noATG cells with and without Dox induction. MTT values normalized to the uninduced (-Dox) condition. Data are shown as mean ± s.d. *P* values (indicated above relevant comparisons) were calculated with two-tailed *t* test (**d**, **g**, **h**) and one-way analysis of variance (ANOVA) with Holm–Sidak tests (**a**, **e**, **f**). Source data for **b**, **c**  provided in Source Data file

The functions of most circRNA remain ambiguous. In particular, the possible functions of virus-encoded circRNAs and those purported to code for proteins remain poorly characterized. To determine the biological functions of circE7, we depleted circE7 in CaSki cells using two doxycycline (Dox)-inducible short hairpin RNAs targeting the circE7 backsplice junction (circE7 sh1/2). After lentiviral transduction of the circE7 shRNA-expressing plasmid, we confirmed the specificity of the circE7 shRNA by RT-qPCR. After Dox induction, both circE7 shRNA resulted in a significant reduction of circE7 levels as assessed both by RT-PCR and northern blotting (Fig. 4a, b). Importantly, we did not note a significant reduction of the linear E6/E7 sequences or levels of the E6*I transcript (Supplementary Fig. 4a–c). Unexpectedly, both RT-qPCR and northern blots suggested that circE7 knockdown actually caused an increase in linear HPV16 E6/E7 transcripts (Supplementary Fig. 4a–b). Next, we tested whether loss of circE7 would impact levels of E7 protein in CaSki cells. Induction of circE7 shRNA 1/2 (sh1/2) decreased levels of endogenous E7 protein by greater than two-fold (Fig. 4c, Supplementary Fig. 4d), demonstrating that circE7 is required for optimal E7 expression in CaSki cells. CircE7 knockdown did not significantly decrease levels of the E6 oncoprotein (Fig. 4c, Supplementary Fig. 4e). Consistent with E7′s established role in transformation, depletion of circE7 resulted in decreased cell proliferation as measured by both cell number and MTT assay (Fig. 4d; Supplementary Fig. 4f-g). CaSki cells expressing circE7 shRNA showed significantly decreased entry into S phase as measured by BrdU incorporation (Fig. 4e, Supplementary Fig. 4h) consistent with a critical role for E7 in overriding Rb’s function in regulating cell cycle progression^25^. Induction of circE7 sh1/2 also significantly inhibited the ability of CaSki cells to form colonies in soft agar (Fig. 4f). To confirm that sh1/2 did not impact CaSki proliferation through off-target effects, a circE7 resistant to shRNA (circResist_WT) was generated by including point mutations in the backsplice junction region while splice site consensus residues were not altered (Supplementary Fig. 5a). To determine whether the protein-coding capacity was required for the function of circE7, a shRNA resistant circE7 lacking start codons was also generated (circResist_noATG) and cloned. CaSki cells were doubly transduced with either vector control, circResist_WT, or circResist_noATG and also the Dox-inducible circE7 sh1/2 vectors (Supplementary Fig. 5a). As expected, while both circResist_WT and circResist_noATG rescued the expression of circE7 by RT-qPCR, only circResist_WT enhanced the expression of the E7 oncoprotein and rendered it resistant to circE7 sh1/2 knockdown (Supplementary Fig. 5c–f). Notably, expression of circResist_WT fully rescued CaSki growth after dox induction of circE7 sh1/2 (Fig. 4g). In contrast, circResist_noATG-expressing cells were able to rescue CaSki proliferation no better than the vector control (Fig. 4h, Supplementary Fig. 5b). In summary, the ability of circE7 to code for the E7 oncoprotein is absolutely essential for the transforming activity of circE7.

**Fig. 5:**
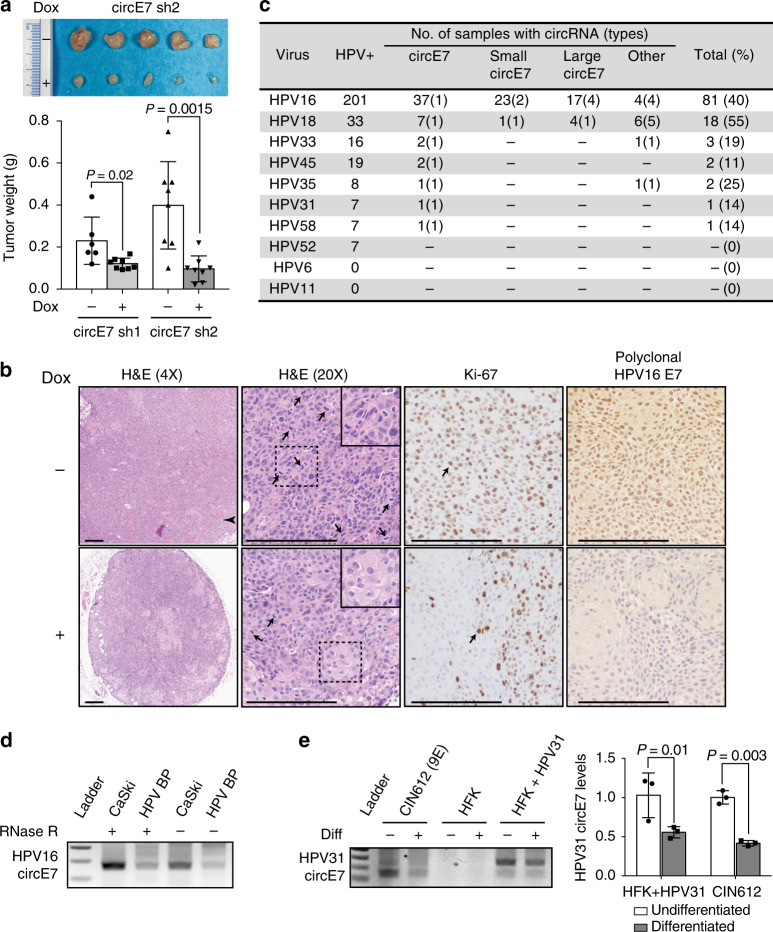
Biological functions of circE7 in tumors and episomal HPV. CaSki cells (4×10^6^), which had been stably transduced with the indicated construct, were xenografted onto the flanks of NSG mice (*n* = 8 per construct). Mice were given water with or without doxycycline (1 mg/mL) as indicated. **a** Image of representative CaSki tumor xenografts dissected from the indicated mice after 21 days (top). Weights of CaSki tumors with or without dox-induced circE7 sh1/2 expression (bottom). **b** Representative images of tumors formed by CaSki xenografts without (top) or with (bottom) doxycycline. Arrowhead indicates an area of invasive tumor. Arrows indicate mitotic figures and Ki-67-positive cells. Dashed box indicates area of detail. Scale bars, 200 μm. **c** TCGA RNA-Seq data (CESC, HNSC) was analyzed with vircircRNA and backsplices with ≥2 reads were tabulated. **d** RT-PCR from CaSki or HPV BP cells that possess integrated or episomal HPV16 genomes with or without RNase R reveals the presence of circE7 in both samples. **e** Human foreskin keratinocyte (HFK), keratinocytes infected with religated HPV31 (HFK + HPV31), or a HPV31 infected cell line derived from a grade II cervical biopsy (CIN612) were induced to differentiate with high calcium. Levels of HPV31 circE7 were assessed by RT-PCR (left) or RT-qPCR (right). Calcium-induced differentiation significantly decreased levels of HPV31 circE7. RT-PCR is representative of 4 independent experiments. Data are shown as mean ± s.d. *P* values (indicated above relevant comparisons) were calculated with two-tailed *t* test (**a**, **e**)

To further evaluate the biological functions of circE7, dox-inducible circE7 sh1/2- expressing CaSki cells were xenografted into immunodeficient mice for 21 days. The induction of circE7 sh1/2, through doxycycline in the drinking water, resulted in significantly smaller tumors (Fig. 5a, Supplementary Fig. 6a-b). Histological analysis of the harvested tumors showed that WT CaSki tumors showed tissue invasion, a high mitotic rate, and pleomorphic nuclei with a high nuclear-cytoplasmic ratio. In contrast, dox-induced CaSki tumors expressing circE7 shRNA were small, well-circumscribed, showed limited tissue invasion, and had a decreased nuclear-cytoplasmic ratio. Immunohistochemical (IHC) evaluation of tumors revealed that circE7 shRNA expressing tumors showed significantly less nuclear staining with the proliferative cell marker Ki-67 than the uninduced control (Supplementary Fig. 6c)^26^. Finally, consistent with western blot analyses, IHC staining revealed markedly less nuclear and cytoplasmic staining for HPV16 E7 oncoprotein expression in CaSki cells after circE7 shRNA induction than the uninduced control. In summary, circE7 is essential for E7 expression and the transformed behavior of CaSki cervical carcinoma cells in vitro and in tumor xenografts.

Given the critical role of circE7 in maintaining the transformed phenotype of CaSki cells, we next determined whether circE7 might be more broadly relevant in human cancers. We analyzed TCGA RNA-Seq data from the mRNA pipeline for cervical squamous cell carcinoma and endocervical adenocarcinoma (CESC) and head and neck squamous cell carcinoma (HNSC) using the vircircRNA pipeline. More than 100 patient tumors with at least two reads matching the same HPV backsplice junction were identified. To ensure the specificity of the identified HPV reads, a control analysis of kidney renal clear cell carcinoma (KIRC), which has been reported to be free of HPV transcripts^27^, yielded no HPV sequence reads or backsplice junctions using identical search parameters. Backsplice junctions were detected from multiple high-risk HPVs, with HPV16 being the most abundant species (Fig. 5c, Supplementary Data 1). Consistent with our preliminary pipeline analysis, multiple species of circE7 were, by far, the most abundant type of backsplice identified in all high-risk HPVs ( >95% of total species). In contrast to the limited detection of circE7 from HPV18 in our in vitro analyses, HPV18+ patient tumors also showed frequent backsplices consistent with HPV18 circE7. As most of the TCGA RNA-Seq samples underwent polyA enrichment, the number of samples and abundance of the circRNA in the TCGA dataset are likely greatly underestimated.

To begin to address whether circRNA expression might have broader relevance to the HPV life cycle, we tested whether circE7 could be detected in HPV BP, a human cervical epithelial cell line that maintains low levels of episomal HPV16. HPV BP contained detectable circE7 (Fig. 5d). In addition, we used a well-established in vitro model of HPV31 infection in which primary human foreskin keratinocytes were transfected with a religated HPV31 genome^28^. HPV31-transfected keratinocytes and an epithelial cell line derived from a grade II cervical intraepithelial neoplastic (CIN) lesion both had detectable circE7 (Fig. 5e). Notably, differentiation of these epithelial cells by high calcium resulted in significant decreases in the levels of circE7 in both HPV31-transfected cells and CIN612 cells^29^. These experiments suggest that circE7 may play a role in the HPV life cycle and could be regulated by keratinocyte differentiation, similar to other transcripts from the HPV genome.

## Discussion

We describe the functional activity of a virus-derived, protein-coding circRNA. Quantitation of northern blots indicated that circE7 represented ~1–3% of total E7 transcripts. Despite its low abundance relative to linear HPV transcripts, circE7 plays an essential role in HPV16′s ability to transform CaSki cells. Post-transcriptional modifications (e.g., m^6^A) and the preferential cytoplasmic localization of circE7 may help explain the striking contribution of circE7 to E7 oncoprotein expression. While HPV16 circE7 could readily be detected through multiple in vitro assays from cancer cell lines and in TCGA RNA-Seq data, HPV18 circE7 was only readily detected in TCGA RNA-Seq datasets. While a higher sensitivity of RNA-Seq compared to in vitro assays could explain this discrepancy, it is likely that species-specific differences in the primary sequence surrounding HPV18 E7 impacts the efficiency of circE7 formation. Alternatively, host cell-specific factors could inhibit HPV18 circE7 formation in the cells and growth conditions that we examined. Overall, our findings are consistent with existing studies demonstrating that circRNA formation is regulated by both cis (sequence-specific) and trans (host cell-specific) factors that regulate the rate of backsplicing events^19^.

Several lines of evidence suggest that circE7 can be translated to generate the E7 oncoprotein. Specifically, mutations that inhibit the formation of circE7 (e.g., splice site mutations, DRACH motif mutations) also prevented translation of the E7 oncoprotein. CircE7, but not a circE7 mutant without start codons, was associated with polysomes. Finally, siRNA specifically targeting the circE7 backsplice junction, but not linear isoforms, prevented E7 oncoprotein production in vitro. Although ‘rolling-circle’ read-through transcription from an exogenous vector could theoretically produce capped, linear mRNAs with ‘false-positive’ backsplice junctions^30^, this would be unlikely to occur in the CaSki cells, which harbor genomically integrated HPV16 genomes. Furthermore, both northern blotting and qRT-PCR strongly implicate circular E7 RNAs as the source of the backsplice junctions in CaSki cells.

The rescue of circE7 knockdown by only shRNA-resistant WT circE7 (circResist_WT), but not a construct without start codons (circResist_noATG), provides strong evidence that the protein-coding capacity of circE7 is essential for its functions in vivo. While the expression of the E7 oncoprotein from circE7 is the most parsimonious explanation for circE7′s transforming properties, it is possible that circE7 may also possess additional activities that promote viral replication and/or host cell transformation. Notably, other linear isoforms encoding E7 (e.g., E6*I) do not fully support E7 oncoprotein translation, despite the fact that such linear transcripts are present even after circE7 knockdown. This suggests that circE7 could play additional roles in promoting linear E7 translation and/or E7 oncoprotein stability. It will be interesting to perform similar studies with other AUG-containing circRNA to determine whether other protein-encoding circRNAs share similar functions as circE7.

HPVs modulate their transcription, polyadenylation, and linear splicing to respond to the differentiation state of the epithelial cells in which they reside. The formation of circRNAs adds another layer of complexity to how HPVs could regulate infection and immune evasion. In the absence of E6, the expression of the E7 oncoprotein upregulates p53 activity and promotes apoptosis^25,31^. Thus, the low translational activity of circE7, in addition to the established stability of circRNAs relative to linear mRNAs, might make them particularly well-suited to promote the fitness of infected cells during latency. Our studies also implicate m^6^A in the regulation of circE7. While a previous study implicated RNA methylation specifically in circRNA translation^20^, the mutation of the abundant m^6^A-consensus motifs in the UTR of circE7 dramatically decreased the efficiency of backsplicing in our assays. Although m^6^A-motifs do not appear to be essential for splicing^32^, we speculate that m^6^A deposition on the nascent circE7 RNA may somehow coordinately regulate backsplicing and translation. While the m^6^A motif sites do not correspond to the binding sites for factors known to regulate HPV splicing^33^, we have not excluded the possibility that the mutations might also directly impact circE7 backsplicing by altering the binding of canonical splicing factors.

While our studies focused on circE7, we speculate that a subset of other viral and protein-coding circRNAs will also have biologically relevant functions. The functional activity of circE7 also suggests important avenues for additional investigation. The factors that regulate circRNA formation and how it differs from the generation of linear RNAs are still poorly understood^34^. The compact size of circE7 may be useful as a template for understanding how backsplicing is regulated. For example, we note that HPV16 circE7 has a unique backsplice 3′ donor (nt 16), but multiple possible upstream backsplice acceptors. While the phenomenon of ‘multiple backsplicing’ has been previously described^11^, the discrete number of defined splice acceptors for circE7 should allow for an analysis of how variable backsplicing is regulated. Moreover, it is interesting that the vast majority of circular backsplices appear to occur in the E6 and E7 early regions of HPV16. We speculate that selective pressure for circRNAs to contain the E7 ORF, a protein essential for the HPV life cycle, drove the formation of circRNA splice sites and ‘signal sequences’ to the region surrounding E6 and E7. It will be interesting to determine whether sequence specific binding activities by both viral and host RNA binding proteins (e.g., E2, E6, SRSFs, hnRNPs) might regulate backsplicing in this region of the genome. In addition, the inverted repeats present in primary sequence of the E6/E7 early region may promote circularization more directly.

More detailed studies on circE7 formation may yield novel insights on how HPVs regulate infection, latency, and tumorigenesis. Finally, the detection of the circE7 backsplice junction may also have clinical implications. Given the stability of circRNA and the importance of the E7 oncoprotein in tumorigenesis, it will be worthwhile determining whether circE7 would be useful as a diagnostic test. As the utility of high-risk HPV testing for cervical cancer screening has already been established, it will be interesting to determine whether circE7 may function as a sensitive marker for the presence of high-risk HPVs and whether its abundance carries any prognostic significance.

## Methods

### De novo circular RNA detection from circular viral genomes

To detect and display circular RNA from RNA sequencing (RNA-Seq) data of viruses with circular genomes, a custom pipeline named vircircRNA was developed (https://github.com/jiwoongbio/vircircRNA). Because the arbitrary linearization of circular genomes causes confusion in distinguishing between back and forward splices, two genomes were concatenated and used as the reference sequence for read mapping. RNA-Seq reads were aligned using Burrows-Wheeler Aligner (BWA, v0.7.15)^35^ with specific options, “-T 19” to reduce minimum score to output and “-Y” to use soft clipping for supplementary alignments. Reads mapped to different positions with soft clipping were extracted as candidate segmented reads by splicing. Canonical GT-AG splice donor-acceptor motifs were used to exclude non-splice reads and define splice breakpoints. Information on strand specificity of sequencing and annotation of genes and promoters were included if available. Splice reads mapped in chiastic order were defined as back-splice reads from circular RNAs. The back-splice junction ratio was calculated by employing the equation:

$$R_{{\rm{back}} - {\rm{splice}}} = \frac{{N_{{\rm{back}} - {\rm{splice}}} \times 2}}{{N_{{\rm{back}} - {\rm{splice}}} \times 2 + N_{5\prime {\rm{forward}} - {\rm{splice}}} + N_{3\prime {\rm{forward}} - {\rm{splice}}}}},$$

where *N*_back−splice_, *N*_5′forward−splice_ and *N*_3′forward−splice_ are the numbers of back-splice, 5′ and 3′ normal-splice junction reads.

### Circular RNA detection from public and TCGA RNA-Seq data

For selected HPV, the genome sequences and the annotations were downloaded from National Center for Biotechnology Information (NCBI) nucleotide database. We used the keywords of “HPV” and “human papillomavirus” to search public sequencing data of HPV from NCBI Sequence Read Archive (SRA) and found 154 RNA-Seq data accessions of cDNA library from 12 projects. For the TCGA data, we downloaded raw sequencing data from cervical squamous cell carcinoma and endocervical adenocarcinoma (CESC, 309 samples), head and neck squamous cell carcinoma (HNSC, 566 samples), and kidney renal clear cell carcinoma (KIRC, 618 samples) from The Cancer Genome Atlas (TCGA). To avoid false mapping, reads (50–51 bp) were mapped onto both HPV genomes and human transcript sequences downloaded from Ensembl (release 92, GRCh38) (https://www.ensembl.org/). Only reads with alignment scores on HPV genomes greater than those on human transcripts were kept for further analysis.

### Cell culture

C-4I cells (gift from Diego Castrillon) were cultured in Waymouth’s and 10% FBS. CaSki cells (ATCC CRM-CRL-1550) were cultured in RPMI and 10% FBS. HeLa (ATCC CCL-2), SW756, and SiHa (gift from Diego Castrillon) cells were cultured in DMEM and 10% FBS. UPCI-SCC154 (ATCC CRL-3241) cells were cultured on Collagen I coated plates in DMEM/F-12 (Gibco, 11320082) supplemented with GlutaMAX (Gibco, 35050061), 10% FBS, 0.1 μg/ml hydrocortisone (Sigma, H0888), and 10 ng/ml EGF (Invitrogen, 10450–013).

### Northern blots

Total RNA from indicated cells was extracted by Qiagen RNeasy. Similar results were obtained using TRIzol (Invitrogen). For cancer cell lines, 20–30 µg of total RNA was treated with RNase R or 8 µg of total RNA used for mock treatment. Similar results were obtained with using rRNA depleted samples (NEBNext) with 4ug of depleted RNA for each sample. For fractionated RNA sample, 4 µg of total RNA per fraction was used. RNA samples were heated in 2× formamide sample buffer and were electrophoresed in 1.5–2.0% formaldehyde-agarose gels in MOPS buffer and transferred onto Hybond N+ membranes (Amersham) with 10xSSC. Probe fragments were generated by PCR using the primers indicated in Supplementary Data 2. PCR products were gel purified (Machery Nagel) and used to generate α-^32^P dCTP-labeled probes using the Random Prime Labeling Kit (Takara/Clontech). Probes were purified over nucleotide purification columns (Zymo Research) prior to hybridization. Hybridization was performed in PerfectHyb buffer (Sigma) at 65 °C overnight and washed according to Hybond’s instructions. Blots were developed on PhosphorImager screens and developed on a Typhoon Imager. Blots were quantited with ImageQuant.

### Circular RNA minigene expression constructs

Circular E7 RNA cDNA were synthesized as gBlock fragments (IDT), PCR amplified, and cloned into pcDNA3.1- vector by *Not*I/*Bam*HI restriction sites. Positive clones were verified by Sanger Sequencing (Genewiz). Constructs were transiently transfected into LentiX-293T (Clontech) cells by Lipofectamine 3000 including the P3000 reagent (ThermoFisher). At 48–72 h post-transfection, cells were harvested for fractionation, RNA preparation, or WB as indicated. For siRNA, 293T cells were plated onto 6-well plates 24 h before transfection to target a density of 30% at the time of transfection. Cells were co-transfected with 60 pmol of the indicated siRNA and 4 μg of the indicated vector without the P3000 reagent. Cells were harvested 68–72 h post-transfection for RNA preparation, WB, or m^6^A IP.

### Western blotting

Whole-cell protein extracts were separated on SDS-PAGE gels, transferred to PVDF membranes and probed with the following primary antibodies: anti-FLAG HRP (1:300, Sigma, A8592), anti-GAPDH (1:1200, Santa-Cruz, sc-32233), anti-HPV16 E6 (1:200, GeneTex, GTX132686), and anti-HPV16 E7 (1:700, GeneTex, GTX133411). Membranes were then incubated with the appropriate HRP-conjugated secondary antibody (1:000, anti-mouse IgG, Cell Signaling Technologies, 7076P2; 1:1000, anti-rabbit IgG, ThermoFisher, G21234) and developed with an ECL system (Perkin Elmer, NEL104001). CaSki cells were harvested and lysed in RIPA buffer (50 mM Tris pH 8.0, 150 mM NaCl, 1% NP-40, 0.1% SDS, 0.5% Sodium Deoxycholate) in presence of 1× protease/phosphatase inhibitor. Cells were incubated on ice with RIPA buffer for 10–15 min. Whole-cell lysate was then cleared by centrifugation at 12,000 × *g* for 15 min. The cleared supernatant was transferred to new tubes, before added Laemmli sampling buffer and boiled for at 95 °C for 10 min.

### RNA isolation, RNase R treatment, cDNA synthesis

Total RNA was extracted from cells using the RNeasy Mini Kit (Qiagen, 74104) and RNase R digested^36^. Similar results were obtained with TRIzol reagent (Sigma, T9424). A concentration of 2 µg of total RNA was incubated with 5U RNase R (Lucigen, RNR07250), 10U murine ribonuclease inhibitor (New England Biolabs, M0314S), 0.5U DNase (Qiagen, 79254), and 1X RNase R buffer for 40 min at 37 °C and then placed on ice. Water was substituted for RNase R in mock reactions. 1 µl of 1 mM EDTA and 1 µl random hexamer (100 µM) were added to the unpurified RNase R digested products and denatured at 65 °C for 5 min. Samples were then placed on ice and reverse transcribed using the Superscript IV RT system (ThermoFisher, 18091050). For standard cDNA synthesis (no RNase R), 1 µg of total RNA was then reverse transcribed using Bio-Rad iScript cDNA synthesis kit according to manufacturer’s instruction.

### End point PCR and RT-qPCR

End point PCR was performed with SapphireAmp (Takara, RR350B) by denaturation at 95 °C for 5 min, followed by 23 cycles at 95 °C 1 min, 62 °C 30 s, 72 °C 1 min, and a final extension step at 72 °C for 7 min for linear products. Cycling conditions for circRNA: were as follows: 95 °C 5 min, followed by 40 cycles of 95 °C 1 min, 62 °C 1 min, 72 °C 2 min, and a final elongation step at 72 °C for 10 min. Cycling conditions for linear mRNA: 95 °C 5 min, followed by 23 cycles of 62 °C 30 s, 72 °C 1 min, 72 °C 7 min, 23 cycles. For end point PCR, cDNA templates were diluted 1:4 in water for linear PCR reactions. For RT-qPCR, cDNA products were diluted 1:20, 2 µL of diluted DNA sample was used as template for real time PCR analysis with PowerUp SYBR Green (Applied Biosystems, A25779). Data were normalized by β-actin as a loading control and then presented as relative normalized expression level.

### Nuclear and cytoplasmic fractionation

Cells grown in 35 mm dishes were trypsinized and centrifuged at 500 × *g* for 3 min at 4 °C. Pelleted cells were washed once with ice-cold PBS, centrifuged again, and resuspended in 250 µl of ice-cold Buffer I [0.5% Triton X-100, 0.32 M sucrose, 3 mM CaCl_2_, 2 mM MgCl_2_, 0.1 mM EDTA, 10 mM Tris (pH 8.0), 50 mM NaCl, 1 mM DTT, 0.04 U/µl RNase inhibitor (ThermoFisher, 18091050)]. After a 15 min incubation on ice, cells were centrifuged at 500 g for 5 min at 4 °C. The supernatant was collected for the cytoplasmic fraction and the pellet was resuspended in 250 µl of Buffer I for the nuclear fraction. RNA was extracted from the cytoplasmic and nuclear fractions using the RNeasy Mini Kit (Qiagen, 74104) and reverse transcribed with Superscript IV RT system (ThermoFisher, 18091050) using random hexamers.

### m^6^A RNA immunoprecipitation assay

pCDNA3.1- empty vector or circE7 expression constructs were transiently transfected into LentiX-293T cells in triplicates. Total RNA was harvested 48–72 h post-transfection. 5 μg total RNA was immunoprecipitated (IP) by 1 μg m^6^A polyclonal antibody (Synaptic Systems#202003)^37^. Immunoprecipitated RNA was then purified over QIAgen RNeasy columns. Purified immunoprecipitated RNA, along with 10% input RNA were then reverse transcribed by Bio-Rad iScript DNA synthesis kit and analyzed by real time PCR. Percentages of m^6^A modified RNA for both circE7 were calculated based on the input reading.

### Polysome purification and RNA preparation

293T cells were plated in 10 cm plates 24 h before transfection to target a density of 40–50% at the time of transfection. Cells were transfected with 10 μg of vector control (pcDNA3.1-), circE7_WT, or circE7_noATG with Lipofectamine 3000 according to the manufacturer’s instructions. After 48 h, the cells were treated with 100 μg/mL cycloheximide in DMSO at 37 °C, and then harvested by trypsinization for polysome profiling. Cells (~5–6 × 10^7^) were lysed in 2 mL of polysome lysis buffer (20 mM Tris pH 7.4, 5 mM MgCl_2_, 100 mM NaCl, 0.1% NP-40, 100 μg/mL cycloheximide) on ice for 15 min, in presence of EDTA-free protease inhibitor cocktail (ThermoFisher), followed by centrifugation at 4 °C for 10 min at 12,000 × *g* to pellet nuclei and mitochondria. The supernatant was then loaded onto a 10–50%(w/v) sucrose density gradient and ultracentrifuged at 20,000 × *g* for 2 h at 4 °C in a swinging bucket TH-641 rotor (ThermoFisher). The fractions were then collected by using BioLogic LP System (Bio-Rad). RNA was extracted from fractions using TriZol LS solution according to manufacturer’s instructions. For end point PCR analysis, 100 ng of RNA from each fraction was used for a standard RT-PCR reaction in 20 μL (ThermoFisher), and then 3 μL of undiluted reaction product was used for end point PCR.

### Lentiviral, doxycycline-inducible shRNA circE7 constructs

Short hairpin RNA (shRNA) sequences specifically targeting the circE7 backsplice were designed using the RNAi Designer Program (https://rnaidesigner.thermofisher.com/). Forward and reverse oligos were annealed in NEB buffer 2, and then phosphorylated and ligated into EZ-Tet-pLKO-Blasticidin vector^38^ (Addgene #85973) using *Nhe*I/*Eco*RI. For shRNA resistant rescue experiments (circResist_WT and circResist_noATG), shRNA resistant constructs were designed and synthesized by Integrated DNA Technology (IDT). Six bases were mutated within the shared shRNA target sequence. All mutations were transversions that were spaced evenly throughout the shRNA targeting sequence thus preventing any residual targeting by non-canonical basepairing or short complementary sequences, respectively. The shRNA resistant constructs were then cloned into a modified pLenti6.3-V5/DEST lentiviral vector. The backbone vector was modified to remove an attR recombination sequence as well as the V5 tag by *Spe*I + *Mlu*I digestion. The linearized vector was then re-ligated by a short oligo containing *Bam*HI restriction site. The constructs were cloned into this modified vector through InFusion cloning with *Bam*HI single digestion. To package lentiviruses, LentiX-293T cells were plated at ~70–80% confluence 12–16 h before transfection. Transfer plasmid, pMD2.G and psPAX2 were then co-transfected into LentiX-293T cells by Lipofectamine 3000 at molar ratio of 1:1:1. Viruses were harvested at 48 and 72 h post-transfection. For lentiviral transduction, CaSki cells were plated into 6-well plate at 50% confluence without any antibiotics. Viral supernatant was then added to the cell in presence of polybrene at final concentration of 8 μg/mL. For single infections (shRNA alone), the cells were then exposed to blasticidin selection 48 h after the intial transduction at a concentration of 15 μg/mL. Cells were selected for ≥7 days before the antibiotic concentration was decreased to 5 μg/mL for maintenance of stable cell lines. For double transductions (shRNA + shRNA resistant circE7), unmodified CaSki cells were lentivirally transduced simultaneously with the Dox-inducible shRNA constructs with a hygromycin selection marker and the shRNA-resistant constructs (circResist) with a blasticidin selection marker. The cells were then selected for more than ≥7 days with 100 μg/mL hygromycin and 15 μg/mL blasticidin. For shRNA expression induction, doxycycline was added to culture medium at final concentration of 1 μg/mL for at least three consecutive days and cells were then subjected to analyses.

### Cell proliferation and MTT cell growth assay

Sixty-thousand (60,000) CaSki cells were plated on Day 0 in quadruplicate RPMI with 10%FBS (doxycycline free) with or without 1 μg/mL doxycycline^26^. Cells were counted daily after Day 2. For MTT-based cell growth rate analysis, 1000 CaSki cells stably transduced with two shRNA constructs were plated into each well of 96 well plates in triplicates for at least 8 days without doxycycline. The first time point was done 24 h after plating, serving as a reference point. After 24 h, doxycycline was added to the rest of plates. The MTT assay was performed daily for 7 days. All the readings from the same well were normalized by the reference point. MTT (Invitrogen#M6494) reactions were performed as recommended by manufacturer.

### BrdU incorporation assay

CaSki cells stably transduced with circE7 shRNA1/2 were induced by 1 μg/mL doxycycline for at least three days. Control cells (no dox) and induced cells were then plated into Nunc 4-well chamber slides (ThermoFisher 154453) in triplicate. Cells were labeled with 10 μM BrdU for 1.5 h. Cells were then fixed by 4% paraformaldehyde at RT for 10 min, followed by 0.1% Triton X-100 permeabilization and 1.5 N HCl DNA hydrolysis. Cells were then probed by O/N incubation with BrdU antibody (1:400, BD BioScience) and stained with Alexa 488-conjugated secondary antibody (ThermoFisher). Samples were then subjected to DAPI nuclear counterstain (Vector Labs). BrdU positive cells were then quantified and data were presented as percentage BrdU positive cells.

### Soft agar colony formation assay

CaSki cells stably transduced with the two doxycycline inducible shRNA constructs were induced by 1 μg/mL doxycycline for 3 days. 0.5% agar, DMEM, 10%FBS was plated as base layer in 6-well plate (35 mm). A total of 10,000 cells of each group were plated in 0.3% agar-DMEM, 10% FBS on top of the base layer in triplicates. Cells were fed with 500 μL RPMI with 10%FBS medium twice a week. Doxycycline (1 μg/mL) was included in all media for induced cells. Cells were then allowed to grow for 28 days before quantification. Colonies larger than 100 μm were scored.

### CaSki xenograft assay

Xenograft assays were performed on eight week-old NOD.Cg-*Prkdc*^*scid*^*Il2rg*^*tm1Wjl*^/SzJ (NSG) (Jackson Laboratory 005557)^39^. Mice were anesthetized and shaved on both flanks and injected subcutaneously with 4 × 10^6^ tumor cells in 200 μl of PBS. Four mice (*n* = 4) were used for each of the four group (sh1/2, −/+ doxycycline). For the Dox-induction group, the cells were pre-treated with 1 μg/mL doxycycline to prime shRNA expression one day before harvesting for injection. Dox-induction animals were given 1 mg/mL doxycycline water, which was changed every 2 days for the duration of the experiment. After 21 days, tumors were dissected and weighed. Tumors were fixed in 4% paraformaldehyde overnight at 4 °C and then moved to 70% ethanol. Immunohistochemical staining of paraffin-embedded tumor tissues was performed using Ki-67 (1:100) and E7 (1:100) primary antibodies. All animal procedures were performed in accordance with institutional guidelines and was approved by the Institutional Animal Care and Use Committee, animal protocol number 2015–101166.

### Reporting summary

Further information on research design is available in the Nature Research Reporting Summary linked to this article.

## Supplementary information

Supplementary Information

Peer Review File

Reporting Summary

Description of Additional Supplementary Files

Supplementary Data 1

Supplementary Data 2

## Data Availability

All data supporting the findings of this study are available from the corresponding author upon reasonable request. The source data for Figs. 2b, 2d, 2e, 2g, 3a, ahd 4b-c are provided as a Source Data file.

## Source data

Source Data

## Code availability

Custom code described in this manuscript is available (https://github.com/jiwoongbio/vircircRNA) under a GNU General Public License, version 3.0 (GPL-3.0).
