# Author Correction: Transforming activity of an oncoprotein-encoding circular RNA from human papillomavirus

**DOI:** 10.1038/s41467-022-30659-z

**Published:** 2022-05-18

**Authors:** Jiawei Zhao, Eunice E. Lee, Jiwoong Kim, Rong Yang, Bahir Chamseddin, Chunyang Ni, Elona Gusho, Yang Xie, Cheng-Ming Chiang, Michael Buszczak, Xiaowei Zhan, Laimonis Laimins, Richard C. Wang

**Affiliations:** 1 Department of Dermatology, UT Southwestern Medical Center, Dallas, TX 75390 USA; 2 Quantitative Biomedical Research Center, UT Southwestern Medical Center, Dallas, TX 75390 USA; 3 Department of Molecular Biology, UT Southwestern Medical Center, Dallas, TX 75390 USA; 4 Department of Microbiology-Immunology, Feinberg School of Medicine, Northwestern University, Chicago, IL 60611 USA; 5 Simmons Comprehensive Cancer Center, UT Southwestern Medical Center, Dallas, TX 75390 USA; 6 Department of Biochemistry, UT Southwestern Medical Center, Dallas, TX 75390 USA; 7 Department of Pharmacology, UT Southwestern Medical Center, Dallas, TX 75390 USA

**Keywords:** Tumour virus infections, Human papilloma virus, RNA metabolism

Correction to: *Nature Communications* 10.1038/s41467-019-10246-5, published online 24 May 2019.

The original version of the Supplementary Information associated with this Article included an error in the Supplementary Data 2 file, in which the sequences of “circE7_WT” and “circE7_noATG” were inadvertently switched. The HTML has been updated to include a corrected version of Supplementary Data 2.

## Supplementary information

Updated Supplementary Data 2

