# Supplementary material for: Transforming activity of an oncoprotein-encoding circular RNA from human papillomavirus
Source: Nat Commun. 2019 May 24;10:2300. doi: 10.1038/s41467-019-10246-5 (PMC6534539; doi:10.1038/s41467-019-10246-5)
Supplement: Supplementary file 1 — Supplementary Information [file 41467_2019_10246_MOESM1_ESM.pdf]

a

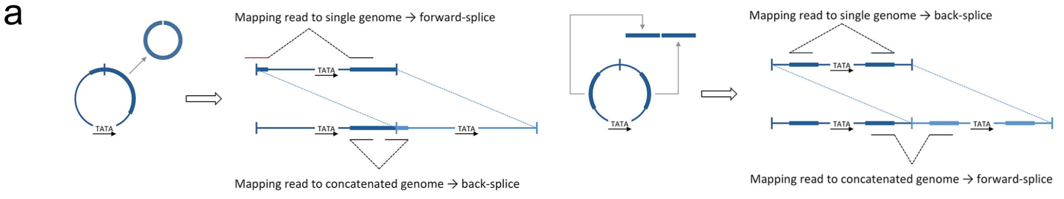

b

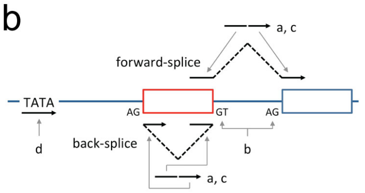

a. Segmented read
b. Canonical splice site (GT-AG)
c. Strand specificity
d. Promoter
e. Mapping topology (back-splice or forward-splice)

c

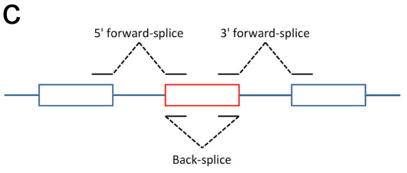

$$R_{back-splice} = \frac{N_{back-splice} \times 2}{N_{back-splice} \times 2 + N_{5' forward-splice} + N_{3' forward-splice}}$$

d

Subtype	Genome accession	Risk
HPV6	NC_001355.1	Low
HPV11	M14119.1	Low
HPV16	NC_001526.4	High
HPV18	NC_001357.1	High
HPV31	J04353.1	High
HPV33	M12732.1	High
HPV35	M74117.1	High
HPV45	KC470258.1	High
HPV52	LC373207.1	High
HPV58	KY225967.1	High

e

Project	Sample	Virus	HPV reads	Backsplice	Genes	Read count	Backsplice ratio
SRP114925	SRS2410540	HPV16	63275	7922-7451	E1^E4,E1,E6,E7	172	0.06541168
SRP114925	SRS2410543	HPV16	75045	7922-7451	E1^E4,E1,E6,E7	78	0.08764045
SRP066090	SRS1159034	HPV16	45950	7922-7451	E1^E4,E1,E6,E7	16	0.05211726
SRP114925	SRS2410540	HPV16	63275	7922-7155	E1^E4,E1,E6,E7	9	0.00719137
SRP066090	SRS1159039	HPV35	80452	883-415	E6,E7,E1	8	0.00431383
SRP095254	SRS1867715	HPV16	86634	7369-7269	E6	7	0.21538462
SRP114925	SRS2410540	HPV16	63275	7922-7568	E1^E4,E1,E6,E7	7	0.00505415
SRP066090	SRS1159031	HPV16	59632	7922-7451	E1^E4,E1,E6,E7	6	0.00585652
SRP114925	SRS2410543	HPV16	75045	7922-7155	E1^E4,E1,E6,E7	5	0.01172333
SRP114925	SRS2410543	HPV16	75045	7922-7568	E1^E4,E1,E6,E7	5	0.01078749
SRP136016	SRS3066238	HPV16	76920	7922-7451	E1^E4,E1,E6,E7	5	0.00840336
SRP066090	SRS1159034	HPV16	45950	7922-7568	E1^E4,E1,E6,E7	4	0.03149606
SRP066090	SRS1159039	HPV35	80452	227-103	E6	3	0.85714286
SRP113560	SRS2599685	HPV16	19910	7922-7451	E1^E4,E1,E6,E7	3	0.08000000
SRP114925	SRS2410543	HPV16	75045	8344-7451	E1^E4,E1,E6,E7	3	0.00640342
SRP114925	SRS2410544	HPV16	68183	7922-7451	E1^E4,E1,E6,E7	3	0.00666667
SRP066090	SRS1159039	HPV35	80452	513-415	E6	2	0.00260756
SRP066090	SRS1159039	HPV35	80452	553-415	E6	2	0.00260417
SRP066090	SRS1159039	HPV35	80452	3575-415	E6,E7,E1,E2,E4	2	0.00260247
SRP095254	SRS1867717	HPV16	76221	7328-7269	E6	2	0.13333333
SRP095254	SRS1867717	HPV16	76221	7369-7269	E6	2	0.13333333
SRP113560	SRS2383444	HPV16	21760	7369-7269	E6	2	0.07142857
SRP113560	SRS2599684	HPV16	23080	7922-7451	E1^E4,E1,E6,E7	2	0.04938272
SRP114925	SRS2410541	HPV16	72054	7922-7451	E1^E4,E1,E6,E7	2	0.00451977
SRP114925	SRS2410542	HPV16	104992	7922-7451	E1^E4,E1,E6,E7	2	0.00192123
SRP114925	SRS2410545	HPV16	94219	7922-7451	E1^E4,E1,E6,E7	2	0.00433839

e

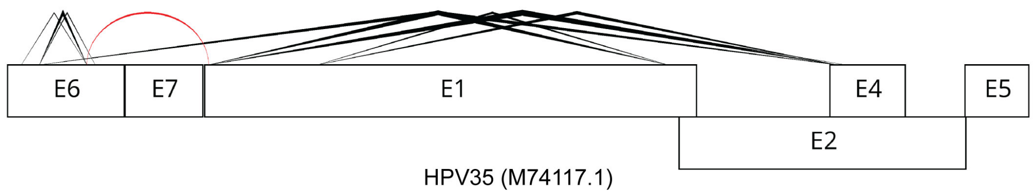

HPV35 (M74117.1)

Supplementary Figure 1. Identification of HPV circRNA. (a) Schematic demonstrating the rationale for concatenation of linearized viral genomes in the vircircRNA pipeline. This strategy allows for the accurate identification of both back and forward splices. **(b)** Criteria employed by vircircRNA to identify splice sites. **(c)** Schematic and formula used to calculate backsplice ratios. **(d)** Type, genome, and risk category of HPV genomes used in vircircRNA analysis. **(e)** Summary of HPV circRNAs identified in SRA datasets including location, genes contained in putative circRNA, read count, and backsplice ratio. **(f)** Diagram generated by vircircRNA summarizing splicing events identified for HPV35. Lines indicate linear splicing; arcs indicate circular splicing; thickness=$\log_2$(read count); red highlights circE7.

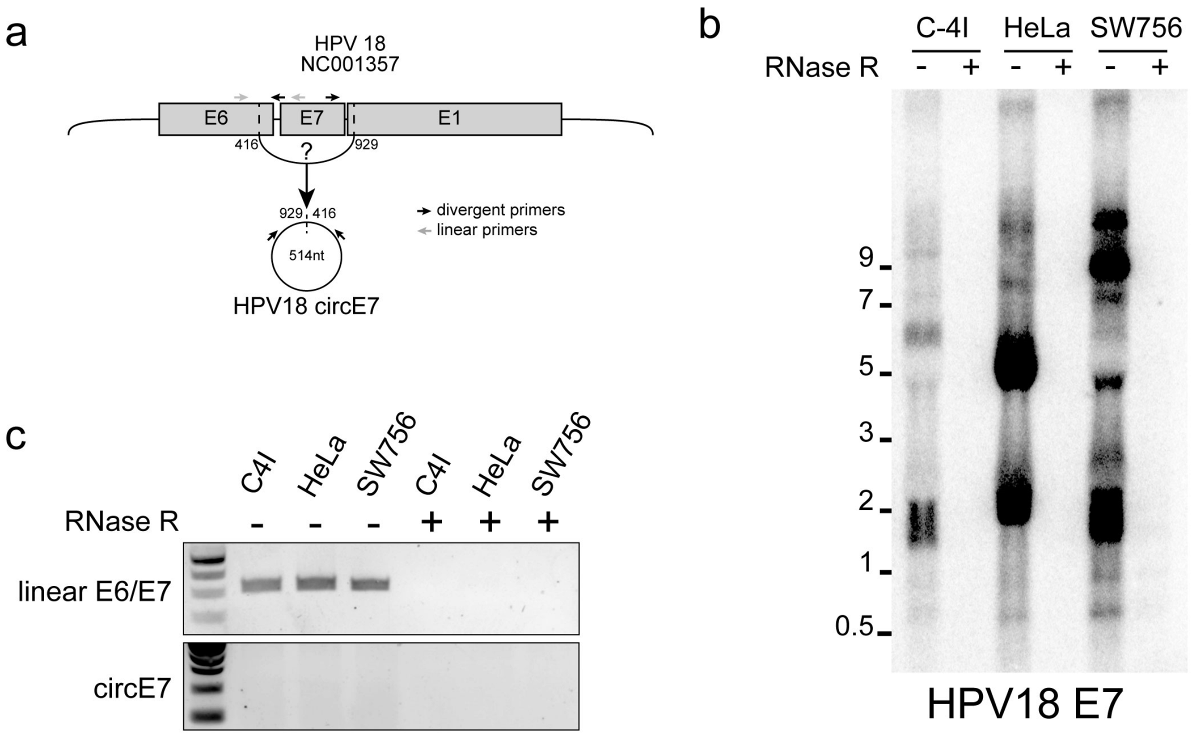

d

Sample	Virus	Backsplice	Genes	Read count
SW-756	HPV18	1225-1136	E1	7
SW-756	HPV18	929-416	E6, E7, E1	5
SW-756	HPV18	233-120	E6	2
SW-756	HPV18	1135-1002	E1	2
SW-756	HPV18	1241-1136	E1	2
SW-756	HPV18	1357-1133	E1	2

Supplementary Figure 2. HPV18 circE7 is rare in HPV18+ cell lines. (a) Predicted size and formation of HPV18 circE7. Splice sites were derived from the analogous splice sites in HPV16. Arrows indicate primers used to detect linear E6/E7 and circE7. **(b)** RT-PCR of random hexamer primed total RNA with or without RNase R treatment reveals loss of linear mRNA. A product consistent with HPV18 circE7 was not detected. **(c)** Northern blot using HPV18 E7 as a probe identifies did not identify RNase R resistant bands in HPV18+ cell lines. Total RNA after mock treatment (8μg) or after RNase R treatment (20μg) from the indicated HPV18+ cell line. **(d)** RNA-Seq from SW-756 revealed the presence of low amounts of several HPV-derived circRNA including a species consistent with HPV18 circE7.

a

HPV16 circE7 miRNA prediction

miRNA Name	Location(s)	RNA-Seq validated
hsa-miR-4251	435	No
hsa-miR-1307-5p	93	Yes
hsa-miR-302f	230	No
hsa-miR-5693	19	No

b

HPV35 circE7 miRNA prediction

miRNA Name	Location(s)	RNA-Seq validated
hsa-miR-4659b-3p	52	Yes
hsa-miR-4659a-5p	52	Yes
hsa-miR-431-5p	171	Yes
hsa-miR-3941	351	Yes
hsa-miR-141-5p	252, 387	Yes
hsa-miR-4423-5p	200	Yes
hsa-miR-195-3p	307	Yes
hsa-miR-16-2-3p	307	Yes
hsa-miR-6784-5p	422	No
hsa-miR-6731-3p	412	No
hsa-miR-8485	350	Yes
hsa-miR-656-3p	305	Yes

c

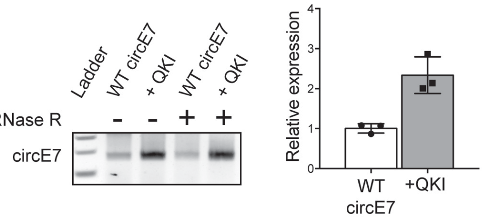

d

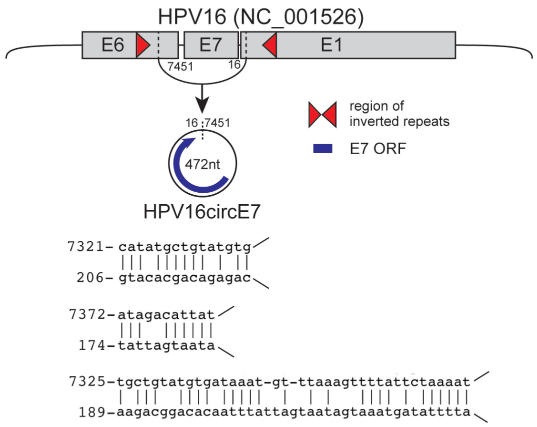

e

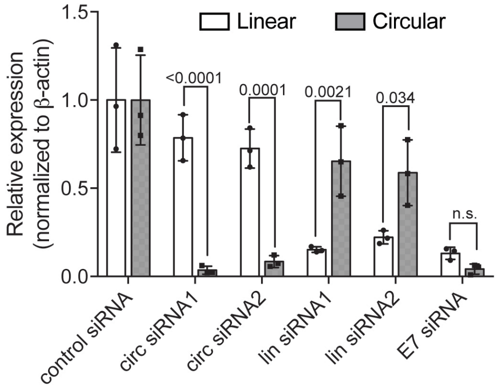

f

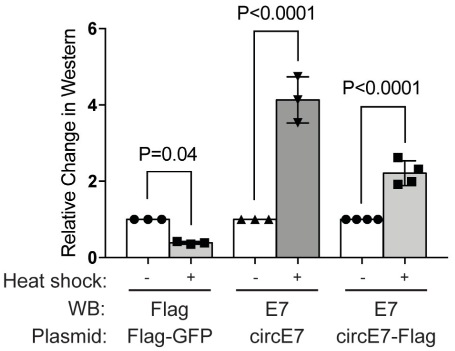

g

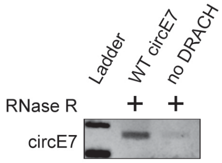

Supplementary Figure 3. Properties of circE7. (a-b) Predicted miRNA binding sites in HPV16 circE7 (a) and HPV35 circE7 (b) (mirDB). No miRNAs are predicted to be conserved between the circE7. **(c)** RT-PCR demonstrates that quaking (QKI) sites increase, but are not required for, circE7 generation from pcDNA3.1-circE7. Endpoint (top) and qRT-qPCR (bottom) from 293T cells reveals that QKI sites increase circE7 formation by >2 fold. Results are representative of 3 independent experiments. **(d)** Schematic indicated several regions of inverted repeats flanking the predicted circE7 product. **(e)** RT-qPCR from 293T cells cotransfected with pcDNA3.1-circE7_FLAG and the indicated siRNAs reveals that siRNA targeting the circE7 backsplice junction (circ) significantly target the circRNA isoform over linear E6/E7; lin siRNAs significantly deplete the linear mRNA; and E7 siRNA depletes both isoforms. **(f)** Quantitation of band density of Western blots for FLAG and HPV16 E7 from 293T cells transfected with the indicated plasmid (Fig. 2D). Values normalized to the control (no heat shock) cells which were not subjected to heat shock (2 hr at 42°C, 2hr recovery). **(g)** RT-PCR demonstrates that DRACH sites in the circE7 are necessary for efficient circE7 generation. Data are shown as mean ± s.d. *P* values (indicated above relevant comparisons) were calculated with two-tailed *t* test (f) and one-way analysis of variance (ANOVA) with Holm-Sidak tests (c,d).

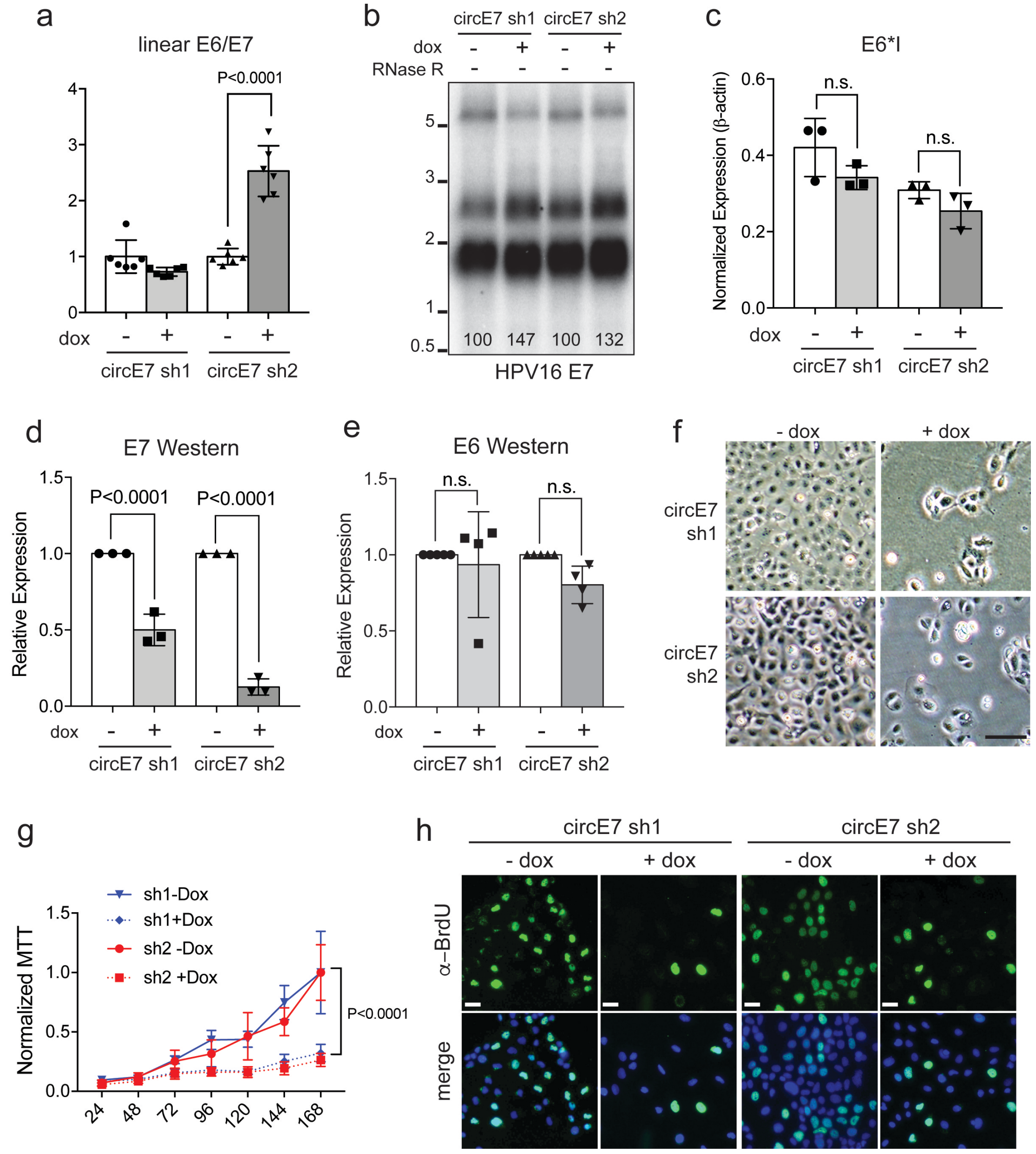

Supplementary Figure 4. Functions of circE7 in vitro. (a) RT-PCR for linear E6/E7 in CaSki with or with induction of circE7 sh1/2. Induction of circE7 sh2 results in a significant *increase* of linear E6/E7 transcripts. (n= 3 independent experiments). **(b)** Northern blot of total RNA (4 μg) from CaSki cells with or without circE7 sh1/2 induction (2 days). Numbers (bottom) indicate quantitation of band density normalized to the uninduced control. **(c)** RT-qPCR for E6*I transcript in CaSki with or with induction of circE7 sh1/2. Induction of circE7 sh1/2 does not significantly change E6*I transcript abundance. **(d)** Quantitation of band density of Western blots for HPV16 E7 from CaSki cells with or without circE7 sh1/2 induction. Values normalized to the uninduced condition. **(e)** Quantitation of band density of E6 from WB from CaSki cells with or without circE7 sh1/2 induction. Values normalized to the uninduced condition. **(f)** Differential interference contrast (DIC) images of CaSki with and without dox induction of circE7 sh1/2 (5 days). Bar, 50μm. **(g)** MTT assay of CaSki circE7 sh1/2 cells with and without doxy induction. MTT values normalized to the uninduced (-Dox) condition. **(h)** Representative images of BrdU and DAPI staining from CaSki with and without dox induction circE7 sh1/2. Bar, 10μm. Data are shown as mean ± s.d. P values (indicated above relevant comparisons) were calculated with one-way analysis of variance (ANOVA) with Holm-Sidak tests.

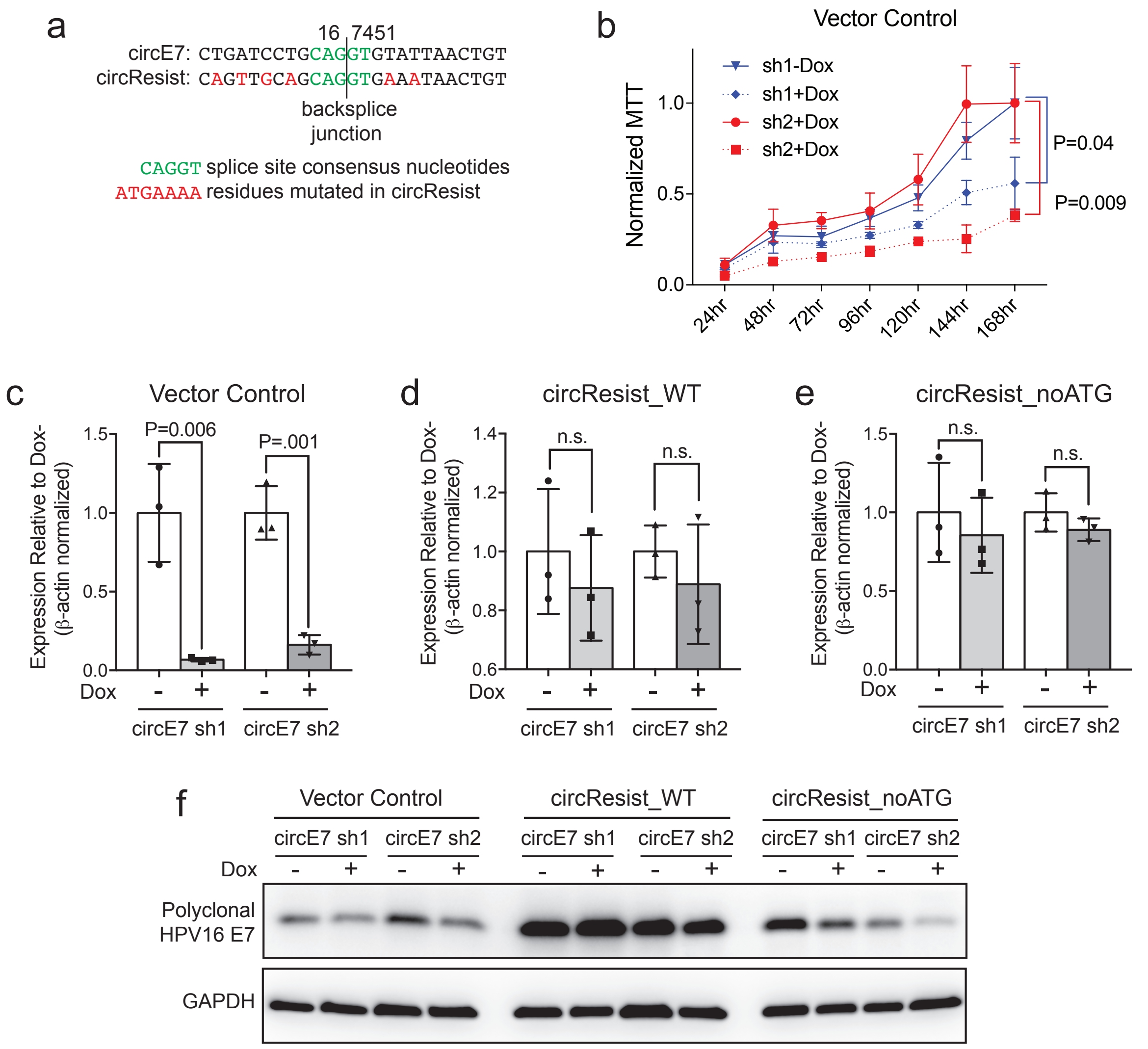

Supplementary Figure 5. Expression of shRNA resistant circE7 (circResist). (a) Schematic of mutations made for shRNA resistant circE7 (circResist). Green text indicates residues important for splice site formation. Red text indicates residues mutated in circResist. **(b)** CaSki were doubly transduced with an empty vector control and circE7 sh1/2. MTT assay of circResist_noATG cells with and without Dox induction. MTT values normalized to the uninduced (-Dox) condition. **(c)** RT-qPCR for circE7 in vector control CaSki with or with induction of circE7 sh1/2. Induction of sh1/2 results in a significant decrease of circE7 transcripts. (n= 3 independent experiments). **(d-e)**. RT-qPCR for circE7 in (d) circResist_WT or (e) circResist_noATG CaSki with or with induction of circE7 sh1/2. Induction of sh1/2 does not result in a significant decrease of circE7 transcripts. The slight decrease in circE7 levels results from targeting of endogenous, but not circResist, circE7 transcripts (n= 3 independent experiments). Data are shown as mean ± s.d. P values (indicated above relevant comparisons) were calculated with two-tailed *t* test (b-e).

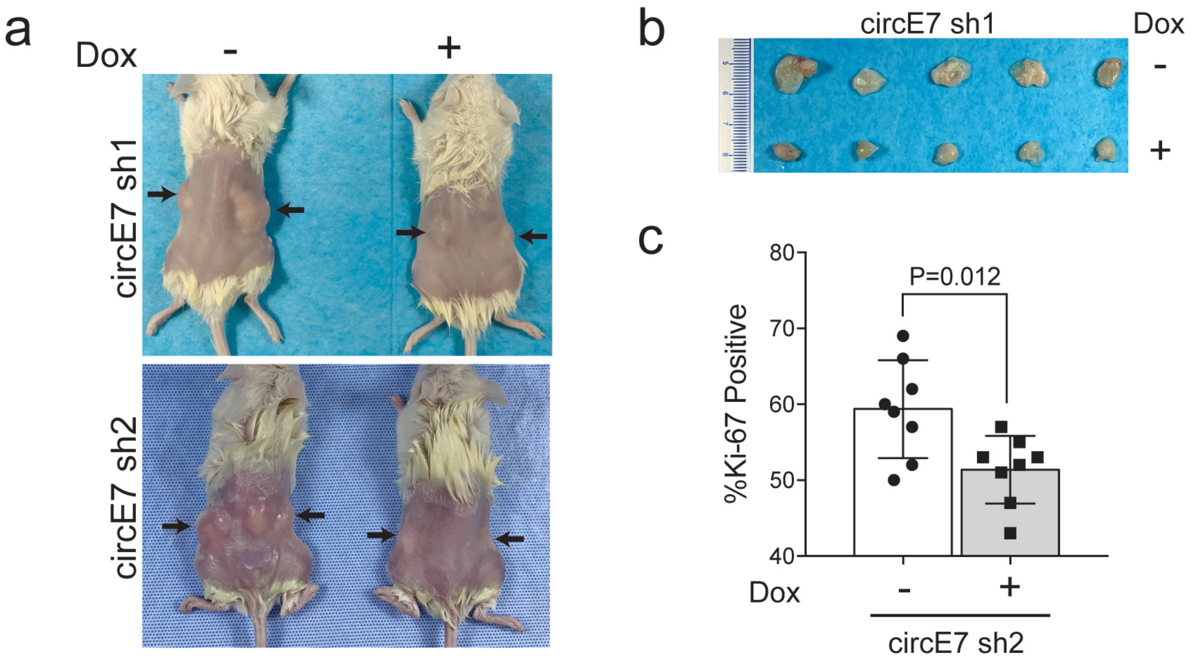

Supplementary Figure 6. Functions of circE7 in vivo. (a) Representative images of NSG mice xenografted with CaSki circE7 sh1/2 with or without dox induction. Arrows indicate tumors. **(b)** Dissected CaSki tumor xenografts from mice that were given water with or without doxycycline (1mg/ml) **(c)** Scoring of Ki-67 staining of CaSki sh2 xenograft tumors with or without circE7 sh1/2 induction. Data are shown as mean ± s.d. *P* values (indicated above relevant comparisons) were calculated with two-tailed *t* test (c).
