# Supplementary material for: Transforming activity of an oncoprotein-encoding circular RNA from human papillomavirus
Source: Nat Commun. 2019 May 24;10:2300. doi: 10.1038/s41467-019-10246-5 (PMC6534539; doi:10.1038/s41467-019-10246-5)
Supplement: Supplementary file 3 — Reporting Summary [file 41467_2019_10246_MOESM3_ESM.pdf]

nature research

Corresponding author(s): Wang, Richard C

Last updated by author(s): Apr 22, 2019

Reporting Summary

Nature Research wishes to improve the reproducibility of the work that we publish. This form provides structure for consistency and transparency in reporting. For further information on Nature Research policies, see Authors & Referees and the Editorial Policy Checklist .

Statistics

For all statistical analyses, confirm that the following items are present in the figure legend, table legend, main text, or Methods section.

n/a	Confirmed	
☐	☒	The exact sample size (*n*) for each experimental group/condition, given as a discrete number and unit of measurement
☐	☒	A statement on whether measurements were taken from distinct samples or whether the same sample was measured repeatedly
☐	☒	The statistical test(s) used AND whether they are one- or two-sided<br>*Only common tests should be described solely by name; describe more complex techniques in the Methods section.*
☒	☐	A description of all covariates tested
☒	☐	A description of any assumptions or corrections, such as tests of normality and adjustment for multiple comparisons
☐	☒	A full description of the statistical parameters including central tendency (e.g. means) or other basic estimates (e.g. regression coefficient) AND variation (e.g. standard deviation) or associated estimates of uncertainty (e.g. confidence intervals)
☐	☒	For null hypothesis testing, the test statistic (e.g. *F*, *t*, *r*) with confidence intervals, effect sizes, degrees of freedom and *P* value noted<br>*Give P values as exact values whenever suitable.*
☒	☐	For Bayesian analysis, information on the choice of priors and Markov chain Monte Carlo settings
☒	☐	For hierarchical and complex designs, identification of the appropriate level for tests and full reporting of outcomes
☒	☐	Estimates of effect sizes (e.g. Cohen's *d*, Pearson's *r*), indicating how they were calculated

Our web collection on statistics for biologists contains articles on many of the points above.

Software and code

Policy information about availability of computer code

Data collection	Microsoft Excel, ImageQuant
Data analysis	ImageQuant, ImageJ, GraphPad Prism, BWA, SAMtools, vircircRNA

For manuscripts utilizing custom algorithms or software that are central to the research but not yet described in published literature, software must be made available to editors/reviewers. We strongly encourage code deposition in a community repository (e.g. GitHub). See the Nature Research guidelines for submitting code & software for further information.

Data

Policy information about availability of data

All manuscripts must include a data availability statement. This statement should provide the following information, where applicable:

- Accession codes, unique identifiers, or web links for publicly available datasets
- A list of figures that have associated raw data
- A description of any restrictions on data availability

All data and custom code that were generated in this study are included in the Figures, Supplementary Information, or github.

Field-specific reporting

Please select the one below that is the best fit for your research. If you are not sure, read the appropriate sections before making your selection.

☒ Life sciences ☐ Behavioural & social sciences ☐ Ecological, evolutionary & environmental sciences

For a reference copy of the document with all sections, see nature.com/documents/nr-reporting-summary-flat.pdf

Life sciences study design

All studies must disclose on these points even when the disclosure is negative.

Sample size	Sample size was determined based on preliminary experiments (pilot experiments from Wang lab) and previously published data.
Data exclusions	No data were excluded
Replication	At least three biological replicates were completed for each experiment.
Randomization	NSG mice use in tumor xenograft experiments were selected randomly.
Blinding	Whenever possible, samples were scored by a research blinded to the treatment conditions (e.g. BrdU staining, colony formation, cell proliferation) samples was performed before scoring data where possible.

Reporting for specific materials, systems and methods

We require information from authors about some types of materials, experimental systems and methods used in many studies. Here, indicate whether each material, system or method listed is relevant to your study. If you are not sure if a list item applies to your research, read the appropriate section before selecting a response.

Materials & experimental systems

n/a	Involved in the study	
☐	☒	Antibodies
☐	☒	Eukaryotic cell lines
☒	☐	Palaeontology
☒	☐	Animals and other organisms
☒	☐	Human research participants
☒	☐	Clinical data

Methods

n/a	Involved in the study	
☒	☐	ChIP-seq
☒	☐	Flow cytometry
☒	☐	MRI-based neuroimaging

Antibodies

Antibodies used	FLAG HRP (1:1000, Sigma, A8592), GAPDH (1:1200, Santa-Cruz, sc-32233), anti-HPV16 E6 (1:200, GeneTex, GTX132686), HPV16 E7 (1:700, GeneTex, GTX133411), m6A (Synaptic Systems, 202003)
Validation	Specificity of HPV16 E6 and E7 antibodies were confirmed against HPV16+ and HPV16- cell lines.

Eukaryotic cell lines

Policy information about cell lines

Cell line source(s)	C-4I, CaSki, HeLa, SiHA, SW756, and UPCI-SCC154 were obtained from the ATCC (some as a gift from another researcher)
Authentication	Cells were used at early passage from the ATCC which provides cell line authentication.
Mycoplasma contamination	Cell lines were negative for Mycoplasma contamination (Sigma LookOut).
Commonly misidentified lines (See ICLAC register)	No commonly misidentified cell lines were used in this study.
