# Supplementary material for: Transforming activity of an oncoprotein-encoding circular RNA from human papillomavirus
Source: Nat Commun. 2019 May 24;10:2300. doi: 10.1038/s41467-019-10246-5 (PMC6534539; doi:10.1038/s41467-019-10246-5)
Supplement: Supplementary file 4 — Description of Additional Supplementary Files [file 41467_2019_10246_MOESM4_ESM.pdf]

Description of Additional Supplementary Files

File Name: Supplementary Data 1
Description: Complete results from vircircRNA analysis of TCGA RNA-Seq data using HPV genomes.

File Name: Supplementary Data 2
Description: Nucleic acid sequences used in this study.
